# Defining the correlation between immune-checkpoint inhibitors-related adverse events and clinical outcomes: a narrative review

**DOI:** 10.3332/ecancer.2021.1314

**Published:** 2021-11-02

**Authors:** Omar Abdihamid, Abeid Omar, Tibera Rugambwa

**Affiliations:** 1 Department of Oncology, Xiangya Hospital, Central South University, Changsha, Hunan 410008, People’s Republic of China; 2 Department of Clinical Oncology and Nuclear Medicine, Faculty of Medicine, Alexandria University, Champlion Street, Alazarita, Alexandria 21131, Egypt; 3 Department of Internal Medicine, Mbeya Zonal Referral Hospital, Mbeya 419, Tanzania

**Keywords:** immune checkpoint inhibitors, adverse events, anti-PD-1, anti-PD-L1, anti-CTLA-4, efficacy, survival, correlation

## Abstract

Immune checkpoint inhibitors (ICIs) have increased modern anticancer armamentarium portfolios, with 15%–60% of cancer patients deriving clinical benefit while others progress, including some occurrences of accelerated progressions. ICIs have also introduced a new pattern of immune-related adverse events (irAEs). Recently, a mechanistic link was proposed in which patients who develop ICIs-related irAEs derive a survival benefit compared to those who do not, suggesting an overlap between toxicities and the treatment efficacy. Identifying predictive biomarkers to optimally identify patients who will benefit from ICIs is a contemporary research area in Oncology. However, the data remains sparse, with only several smaller studies showing a plausible direct proportionality of a therapeutic effect across tumours. In contrast, the overall survival and progression-free survival rate depend on the tumour type, degree of toxicities, duration of exposure, affected system/organs and inherent patient characteristics. Furthermore, the occurrence of irAEs appears to be more associated with a clinical benefit from programmed death 1 and programmed death-ligand 1 inhibitors than anti-cytotoxic T-lymphocyte-associated antigen 4. Several questions remain unanswered, including the association between survival benefit and specific type of organ system toxicities, toxicity grade, if the benefit is entirely due to immortal-time biases (ITBs), presence of patients confounding comorbidities like autoimmune diseases, and finally, immune heterogeneities. Considering ITB represents a key element in interpreting these studies since patients with precipitated death or with an earlier disease progresses rarely develop irAEs; in fact, such patients have not stayed in the study long enough to experience such irAEs. Conversely, patients that stayed in the study for a longer period have a higher risk of developing irAEs. Landmark analysis is key in these studies if a real association is to be found. Overall response and disease control rates are mainly higher in those who develop irAEs due to immune activation. So, this review aims to summarise the evidence from key studies that addressed this important clinical question.

## Introduction

Our immune system remains our first line of defence in any disease, including cancer. Harnessing it by enhanced immune activation while maintaining an equilibrium of self-tolerance is what modern science continues to achieve.

Cancer cells harbour mechanisms to evade immune surveillance by hijacking the physiological immune negative regulators, posturing as a ‘self’ cell, and eventually evading immune distraction. Immune checkpoint inhibitors (ICIs) are a class of anticancer drugs known as monoclonal antibodies that target receptors like anti-programmed death 1 (anti-PD-1), anti-programmed death-ligand 1 (anti-PDL-1) and the anti-cytotoxic T-lymphocyte-associated antigen 4 (anti-CTLA-4). By inhibiting these innate negative T-cell regulators, the immune system is activated, and its ability to recognise and kill tumour cells is enhanced [1–3]

Immunotherapy is currently considered the ‘fifth pillar’ of cancer treatment, joining the ranks of chemotherapy, radiotherapy, surgery and targeted therapy. Over the years, several ICI agents have been approved, improving the overall survival (OS) of many cancer patients (Table 1). Amongst the approved ICI agents, anti-PD1 agents have the largest indication across several tumours, including metastatic non-small cell lung cancer (NSCLC) [4], advanced head and neck cancer (HNC) [5], advanced melanoma [6], genitourinary tumours and others [7, 8].

Randomised clinical trials in immuno-oncology include the Keynote 045 trial [9], which showed prolonged OS and preservation of good quality of life (QoL) scores in patients with urothelial cancers (UCs).

The Checkmate 141 trial [5] looked at Nivolumab as a first-line therapy in patients with relapsed or metastatic HNC, and it showed an improvement in progression-free survival (PFS) and QoL.

The Checkmate 214 phase 3 trial [10] looked at renal carcinoma patients receiving either double ICI agents or sunitinib and showed a similar positive outcome in both OS and improved QoL scores in the ICI group.

While there is a well-documented clinical benefit from ICIs, the activated immune system causes several off-target and bystander effects to normal tissues, leading to the so-called immune-related adverse events (irAEs) [11]. The similarities between antigens presented on normal tissues and tumour cells are the leading hypotheses for developing these adverse events [12]. Similarly, the aetiology of irAEs is akin to those that promote the anti-tumour effect and mainly involves the expansion of the T cell repertoire. Also, ICIs can affect clonal B cell responses, thus inducing autoantibody production [13].

Some irAEs can be subtle and tolerable, while others are rare but life-threatening. irAEs can result in an array of systemic, multi-organ inflammations like liver, thyroid, pituitary, colon and lungs [14].

Studies show that ICIs have lower nausea, vomiting, anorexia, fatigue, diarrhoea, neuropathy, haematologic toxicities and treatment discontinuation rate than conventional chemotherapy. Conversely, the signature irAEs like skin rash, pruritus, colitis, pneumonitis, transaminitis and endocrine toxicities are more common with ICIs than chemotherapy. Lastly, ICIs are overall better tolerated than chemotherapy [15].

Although most irAEs are identified during pre-clinical development, the spectrum, timing and outcomes were only recently investigated through real-world, large-scale pharmacovigilance analyses like the spontaneous reporting systems that looked at the real-time monitoring of these drugs’ safety profiles [16]. The term ‘immunovigilance’ and disproportionality analyses (DAs) have also been proposed. DAs are now the most common epidemiological approach for postmarketing safety assessment and documentation in many ICI agents [17].

There is growing evidence suggesting a potential association between irAEs and clinical benefit in some cancer types [18], and in fact, several reports have shown that irAEs are not only associated with ICI efficacy but also precede response [19–21]. However, a possible bias exists in which responders benefit from long-term ICI therapy and are therefore exposed to a higher risk of developing irAEs throughout time [22].

The main bias in the association between irAEs and clinical outcomes is the immortal time bias (ITB); defined as bias arising from methodologically wrong analyses of time-dependent events in survival analyses [23].

Considering ITB represents a key element in interpreting these studies since patients with precipitated death or with an earlier disease progresses rarely develop irAEs; in fact, such patients have not stayed in the study long enough to experience such irAEs. Conversely, patients that stayed in the study for a longer period have a higher risk of developing irAEs.

Furthermore, despite several studies providing interesting data, this association should be interpreted with caution because the majority of these studies did not report or adjusted for the effect of ITB in their survival analyses [24].

Landmark analysis is the recommended solution to such biases since it splits the follow-up period at a given time point. Patients groups are then defined by time-dependent development of irAEs, such as before and after the landmark, with clinical outcomes only considered if occurring after the landmark [23].

## The spectrum of irAEs

Cutaneous toxicities are the most common irAEs resulting from ICI therapy and often present as a distinct pruritic maculopapular rash, commonly seen on the trunk and the limbs, exacerbated by underlying psoriasis. A grade 3 or 4 (G3, G4) with a bullous appearance akin to Stevens–Johnson syndrome is seen in severe cases, especially in patients with underlying autoimmune disease [21, 25, 26].

Gastrointestinal symptoms such as abdominal pain and diarrhoea secondary to colitis are classic ipilimumab-related irAEs that manifest as loose stool, abdominal pain, haematochezia and fever. Interleukin-6 productions can cause an attack on enterocytes by the activated T-cells, and if left unrecognised, it can lead to intestinal perforation. Hepatic involvement usually manifests as asymptomatic hepatitis with transaminitis. Acute pancreatitis can present with abdominal or back pain with nausea and vomiting or can be more severe by causing autoimmune destruction of the pancreatic islet cells. Such patients are usually asymptomatic until they present with full-blown diabetic ketoacidosis [27].

Endocrine irAEs can be insidious in onset and usually present with hypophysitis with diminished downstream thyroid and adrenal steroid production, or occasionally with severe headache as the pituitary becomes acutely inflamed [28].

Renal toxicity is typically detected as high serum creatinine levels, which usually prompt the discontinuation of ICIs. Pneumonitis classically presents with a dry cough, dyspnoea with or without hypoxaemia. Ocular toxicities can present with visual symptoms such as blurry vision and retinitis or even proptosis resulting from the inflammation of extraocular muscles.

Neurologic toxicities are fatal irAEs and usually involve the brain, peripheral nervous system, leptomeninges or even autoimmune destruction of the autonomic nervous system. The antiviral response resulting from the use of ICI agents can result in encephalitis.

The endogenous expression of CTLA-4 on the pituitary can also lead to hypophysitis. Musculoskeletal toxicities usually include myalgias, arthralgias with elevated muscle enzymes signifying significant muscle destruction [26, 27].

**Figure 1.**
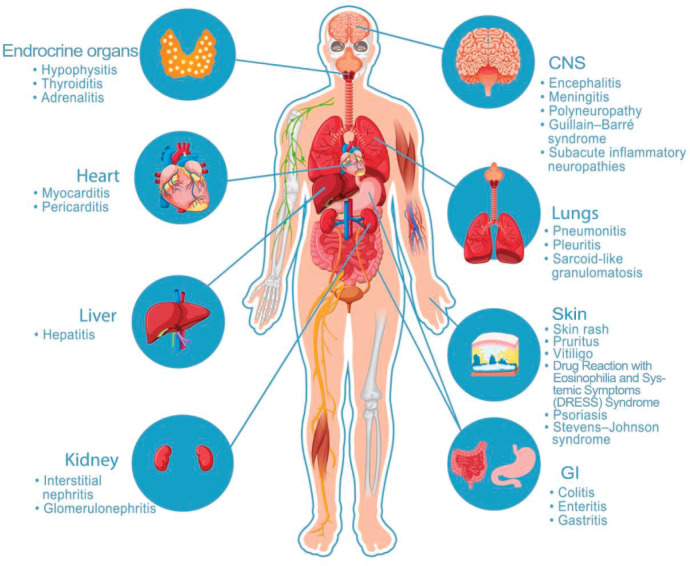
Immunotherapy-related anatomic based irAEs. Anatomic based ICIs related adverse events.

Cardiotoxicity, which can present as fulminant myocarditis, is one of the rare but catastrophic irAEs. Myocytes express shared antigens with the tumours, which can lead to autoimmune distraction. It usually mimics heart failure symptoms and can also present with arrhythmias and conduction abnormalities. These patients are usually managed with joint help from cardiologists, even as the cardio-oncology sub-speciality continues to become popular [29, 30]. A pictorial view of the organ system-based irAEs is shown in Figure 1.

## Incidence

Data emerging from randomized clinical trials (RCTs) shows that the frequency and the incidence of irAEs usually depend on the ICI agent used, duration of exposure, dose and patient’s risk factors profile. On the other hand, the kinetics and the timing of irAEs are dependent on the affected organ system [21]. A higher incidence of G1 to G5 toxicities is usually common in anti-CTLA-4, moderate with PD-1 class and lower in PD-L1 agents. Intriguingly, different tumour types had different tissue type inflammation resulting from ICI use. For example, colitis was mostly found in melanoma patients [31], whereas pneumonitis was more common in lung cancer patients [32].

In a review of 125 RCTs, 75 different irAEs, including fatigue (18.3%), diarrhoea (9.5%) and pruritus (10.6%), have been reported. Also, two in every three patients experienced at least one G3 toxicity, citing the true incidence and impact of irAEs [33]. A systematic review of 35 immunotherapy RCTs reported the true incidence of IrAEs as follows: PD(L)-1 inhibitors (14%), ICI combinations (55%), ICI-chemotherapy combo (46%) and CTLA-4 inhibitors (34%). Grade 3 or more IrAEs were common, especially in ICI combination therapies. Thirty-two (91%) RCTs were performed in the metastatic or locally advanced stage, whereas only three (9%) trials were conducted on neoadjuvant or adjuvant settings [34].

ICI monotherapy versus combined ICI is associated with fewer irAEs. A meta-analysis showed that the ICI combo significantly increased the development of any-grade irAEs, including colitis, pneumonitis, hypothyroidism, hypophysitis, amongst others [35]. Similarly, an analysis of 18 studies with 2,767 patients across cancer types showed that combination ICI therapy was associated with a higher risk of all-grade irAEs (Response rate (RR): 1.07; 95% confidence interval (CI): 1.03–1.11) and markedly greater risk of G3 or higher adverse events (RR: 2.21; 95% CI: 1.57–3.10) compared to monotherapy ICI [36].

A detailed study that queried irAEs from the World Health Organization databases (VigiBase) found 613 ICI-related fatal toxic events from 2009 to 2018. Amongst these fatalities, 193 deaths from anti-CTLA-4 agents, mostly from colitis (135 cases), while fatalities from pneumonitis (333), hepatitis (115) and neurotoxic effects (50) were associated with anti-PD-1/PD-L1 use. Similarly, deaths from ICI combos were frequently from colitis (25 cases) and myocarditis (32 cases) [30].

Retreatment with ICI after a clinically significant previous irAE is common. A multicentre retrospective study of 499 patients identified ICI patients with renal cell carcinoma (RCC) in which 80 patients developed severe irAEs warranting treatment cessation, 36 of whom were able to be restarted on an ICI agent and 44 permanently discontinued. Time to initial irAE was similar between the two groups (2.8 versus 2.7 months; *p* = 0.59) [37].

Similarly, in a retrospective study of 80 patients with metastatic melanoma who received a combined CTLA-4 and a PD-1 inhibitor, patients who developed irAEs warranting treatment discontinuation were later rechallenged with a single-agent PD-1 inhibitor. The rates of toxicities amongst patients who resumed single-agent anti-PD-1 were notably lower (for example, G3/4 colitis declined to 3%, and G3/4 hepatitis declined to 7%) [38].

A review of 38 RCTs comprising 7,551 patients on ICI therapy evaluated the incidence of endocrine irAEs and found a higher incidence of hypo and hyperthyroidism, especially in patients receiving combined ICI therapy (OR: 3.81; 95% CI: 2.10–6.91; *p* < 0.001). Moreover, PD-1 inhibitors, in particular, were associated with hypothyroidism as compared to CTLA-4 inhibitors (odds ratio (OR): 1.89; 95% CI: 1.17–3.05; *p* = 0.03) [39].

Interestingly, it is also common to diagnose irAEs even after the discontinuation of ICI agents. In a retrospective review of 64 patients with metastatic melanoma treated with Nivolumab plus Ipilimumab, 31 patients experienced early toxicity warranting discontinuation of treatment, and 4 of these (13%) further developed a significant irAEs 4 months after treatment cessation. Therefore, both clinicians and patients should be aware of this trend [40].

Therefore, it is crucial to anticipate the occurrence of irAEs and educate patients on the risks. Weber *et al* [41] conducted a pooled safety data analysis from four studies consisting of 576 patients with irAEs of any grade. They found most of these events occurred within the first 4 months of therapy, and skin was the commonly involved site, followed by the gastrointestinal and the endocrine system. The authors also noted some toxicities occurred as late as day 60.

On the general safety profiles of ICI agents, a head-to-head comparison of 36 phase 1 and phase 2 clinical trials that enrolled 15,370 patients found that the safety ranking of ICIs as follows: Atezolizumab (pooled incidence 66.4%, probability 76%), Nivolumab (71.8%, 56%), Pembrolizumab (75.1%, 55%), Ipilimumab (86.8%, 55%) and Tremelimumab (not applicable (NA), 54%). Atezolizumab had the overall best safety profile, while Nivolumab had the best safety data in lung cancer [42].

Lastly, tumour and class-specific irAEs are an unresolved and controversial topic. While anti-CTLA-4 agents are believed to be more toxic than anti-PD-1/PD-L1 drugs, a definitive comparison is lacking due to their different therapeutic uses. However, others have speculated that anti-PD-L1 drugs could be less toxic than anti-PD-1 agents due to the preservation of PD-L2 signalling [43].

## Tumour-specific irAEs and clinical outcomes

A mechanistic link was proposed by the investigators of a prospective cohort study in which 73 NSCLC patients treated ICI showed that there was shared cancer and tissue antigens and T cell infiltration in both the lung tumour and the skin, suggesting an overlap between the toxicities and the efficacy [44].

In a similar study of 559 NSCLC patients, the incidence of irAEs was 41.3% (231 patients), and 50 patients (8.9%) developed grade 3 or 4 events. Using a landmark analysis at 6 weeks, the authors confirmed that irAEs of any grade were an independent predictor of higher overall response rate (ORR), longer OS and more prolonged PFS [45].

A study of 99 Phase III oncology RCTs found both toxicity and efficacy were higher amongst the treatment arm than the control (all grade toxicities were 3.5 times higher in the treatment than the control arm, *p* < 0.001; mean OS of 18.6 versus 16.9 months; *p* < 0.001; mean PFS of 9.1 versus 7.1 months; *p* < 0.001, respectively). Notably, the authors concluded that, across the RCTs, irAEs were strongly associated with higher PFS in ICI patients versus controls (*p* < 0.001), but not OS (*p* = 0.44) [46].

A systematic review looking at the emerging evidence on the association between IrAEs and clinical outcomes highlighted the existence of a plausible direct proportionality of a therapeutic effect across ICI agents and solid tumours [47].

A meta-analysis of 30 studies that enrolled 4,324 patients who received ICI, those who developed irAEs had a reduced risk of death (hazard ratio (HR) = 0.49; 95% CI: 0.38–0.62; *p* < 0.001), and the development of irAEs conferred a reduced risk of progression (HR = 0.51; 95% CI: 0.42–0.64; *p* < 0.001). The authors believe these findings could be related to strong immune activation [48].

In a large safety and efficacy data analysis of 1,783 patients receiving Avelumab from the Merkel 200 trials and the JAVELIN Solid Tumor, 16% (295 patients) developed irAEs. A time-dependent Cox model analysis found a reduced risk of death in patients with irAEs than those without (HR: 0.71; 95% CI: 0.59–0.85) [49].

In a multicentre pooled analysis of 531 patients with metastatic NSCLC treated with Nivolumab as a second line therapy, 33.0% (173 patients) developed irAEs and had a significantly longer OS (14.9 versus 7.4 months; HR: 0.66; 95% CI: 0.52–0.82); *p* < 0.001 as compared to patients with no irAEs and a PFS (6.1 versus 3.1 months; HR: 0.68; 95% CI: 0.55–0.85); *p* = 0.001. The authors also noticed a negative impact on patients’ prognosis resulting from treatment discontinuation due to irAEs; median OS (3.6 versus 17.6 months; HR: 2.61; 95% CI: 1.61–4.21); *p* < 0.001, PFS (2.3 versus 6.6 months; HR: 1.74; 95% CI: 1.06–2.80); *p* = 0.02 [50].

ICI has revolutionised the treatment landscape and improved survival in metastatic melanoma. The onset of specific irAEs like vitiligo has been investigated in different studies. In a multivariate analysis, irAEs were associated with improved PFS (HR: 0.47; 95% CI: 0.26–0.86); *p* = 0.016, and a better OS; (HR: 0.39;95% CI: 0.18–0.81); *p* = 0.007. Vitiligo was associated with a moderate improvement of OS compared with other irAEs (*p* = 0.061) [51, 52]

In a similar melanoma study in the Chinese population of 93 patients who received ICI therapy, frequent irAEs were pruritus, vitiligo, rash and fatigue. The median onset of irAEs was 6.1 weeks. Both disease control rates (DCRs) and ORR were higher in the irAEs group than those without (*p* = 0.004 and *p* = 0.003, respectively) [53].

Furthermore, apart from the established impact of cutaneous irAEs on OS of melanoma patients, gastrointestinal irAEs, especially diarrhoea, which usually limits ICI treatment, was paradoxically associated with improved OS and PFS in metastatic melanoma patients (HR 0.56; 95% CI: 0.41–0.76; *p* < 0.01) [31].

Thyroid irAEs induced by Nivolumab in a cohort of 200 Japanese patients found that patients with overt thyroid uptake as depicted by fluorodeoxyglucose-positron emission tomography (FDG-PET) had a high incidence of irAEs but with an OS benefit (16.1 versus 13.6 months; HR: 0.61; 95% CI: 0.39–0.93) [54].

A retrospective pan-cancer analysis study, albeit from a single centre looking at 212 patients with either metastatic or recurrent RCC, melanoma, NSCLC and gastric cancer (GC) who received ICI therapy, showed that 108 patients had solitary organ irAEs, while 42 developed multiple site irAEs. Generally, the OS was longer in patients with any category of irAEs than those without irAEs (28.1 versus 12.7 months; HR: 0.49; 95% CI: 0.33–0.73; *p* = 0.0004). Also, patients with multiple site irAEs had a markedly longer OS than those with single site irAE (42.3 versus 18.8 months; HR: 0.473; 95% CI: 0.346–0.647; *p* < 0.0001) [55].

In a similar anatomical based irAEs study, a meta-analysis that included 4,971 patients from 30 studies found both OS and PFS benefits from ICI therapy as compared to those without irAEs (PFS: HR: 0.52; 95% CI: 0.44–0.61; *p* < 0.001; OS: HR: 0.54; 95% CI: 0.45–0.65; *p* < 0.001). Further subgroup analyses found that dermatological irAEs (OS: HR: 0.45; 95% CI: 0.35–0.59; *p* < 0.001), endocrine irAEs (OS: HR: 0.52; 95% CI: 0.44–0.62; *p* < 0.001) and low-grade irAEs (OS: HR: 0.57; 95% CI: 0.43–0.75; *p* < 0.001) equally yielded same results [56].

Although cutaneous irAEs like vitiligo confers a clinical efficacy in melanoma, the benefit in other cancers remains unknown. A retrospective 6-week landmark analysis of 155 NSCLC patients who received either Pembrolizumab or Nivolumab monotherapy showed that the median time to rash onset was 6.4 weeks. The ORR was higher in patients with cutaneous reactions (57% versus 19%; *p* < 0.001) and a median PFS of 12.9 versus 3.5 months. Multivariate analysis identified rheumatoid factor positivity as an independent predictor of cutaneous irAEs [57].

A similar retrospective analysis of 40 NSCLC patients on Nivolumab found a better RR in those with skin toxicity (42%) than those without (7%) [12].

Rheumatological irAEs are not commonly reported as signature irAEs in patients receiving immunotherapy. Kostine *et al* [58] looked at the impact of rheumatologic side effects in all ICI agents and irAEs sites on ORR. The ORR was higher in patients with rheumatologic side effects (85.7% versus 35.3%; *p* < 0.0001) compared to their counterparts [58].

The association of irAEs and clinical outcomes in NSCLC patients with existing brain metastasis who received ICIs is also not well studied. A small retrospective review of 63 patients found that any grade irAEs were associated with longer OS (21 versus 10 months; *p* = 0.004) and delayed treatment failure (14 versus 5 months; *p* = 0.001), especially in patients with high PD-L1 expression [59].

ICI-related pneumonitis (ICIP) is a common and potentially life-threatening irAE in NSCLC patients. A real-world cohort study looked at the risks of developing ICIP in NSCLC patients who received prior radiotherapy and are currently on ICI therapy. Expectedly, the study found prior radiotherapy was not only associated with a higher risk of ICIP but was an independent prognostic factor for low survival in NSCLC patients [60].

Immunotherapy data in GC is not as robust, and the survival benefit from ICI has been modest. Nonetheless, a multicentre study of 76 GC patients who received Food and Drug Administration (FDA) approved ICI agents showed irAEs were associated with a better outcome in PFS and OS (PFS: 3.9 months versus not reached; HR: 0.13; 95% CI: 0.05–0.3; *p* < 0.001; OS: 7.4 months versus not reached; HR: 0.11; 95% CI: 0.03–0.36; *p* < 0.001 [61].

On the other hand, UCs have received more ICI agents FDA approvals than many other cancers. The occurrence and the spectrum of IrAEs corresponded with higher efficacy, higher ORR (52% versus 16%; *p* < 0.01), better PFS (11.0 versus 3.6 months; *p* < 0.01) and OS (13.1 versus median not reached) as compared to those without IrAEs [62]. In another study of UC patients, those who developed irAEs had an ORR of 33.0% versus 8%, and at 15 months median follow-up, all the patients with irAEs were still alive, compared to only 38% of their counterparts [63].

Metastatic RCC patients receiving ICI therapy who developed irAEs had a median OS of 35.9 versus 26.5 months; HR: 0.376; 95% CI: 0.179–0.792; *p* = 0.010 [64]. Similarly, Ishihara *et al* [65] reported a PFS (13.1 versus 4.87 months; HR: 0.25; 95% CI: 0.11–0.56) in RCC patients with any grade of irAEs versus those without.

Immunotherapy is also approved for metastatic HNC [66]. A similar benefit on RR, PFS and OS has been reported in patients receiving ICI agents who developed irAEs [67]. A single-centre study of 89 patients with relapsed or metastatic squamous cell HNC treated with Nivolumab showed that the median OS was significantly longer in patients with irAEs than those without (17.9 versus 6.3 months; log-rank *p =* 0.004). The authors also reported a median PFS (10.2 versus 2.8 months) in those with versus without irAEs [68].

Although not commonly reported, ICI therapy-related pyrexia was associated with a longer PFS in one study (6.4 versus 2.1 months; *p* = 0.26) in patients with advanced NSCLC. Similarly, a better ORR trend was also seen in those who developed pyrexia (65% versus 35%; *p* = 0.29) [69].

Apart from the common ICI monoclonal antibody-based drugs, monocyte-derived vaccines or dendritic cells (DC) loaded with tumour-associated antigens gp100 are also part of the immuno-oncology armamentarium. In a retrospective analysis of advanced melanoma patients vaccinated with DC vaccines, 84% of the patients developed irAEs, mostly injection site reactions (50%) and flu-like symptoms (67%), and both correlated with immunologic and clinical outcomes (both *p* < 0.001) [70].

Further compounding the association between the occurrence of irAEs and efficacy conundrum is the common use of steroids to counteract adverse events in patients receiving ICI therapy. In a study by Riudavets *et al* [71], Prednisone has been found to significantly affect median OS in patients receiving more than 10 mg a day than those with less than 10 mg (6 versus 15.9 months; *p* < 0.001) [71].

Conversely, a retrospective analysis of 157 patients with different tumour types assessed the effects of irAEs and steroid use on PFS. Forty-five of these patients developed irAEs, in which 21 patients received steroids. Development of irAEs and steroid use was found to improve PFS by Kaplan–Meier estimate. Also, multivariate regression showed that irAEs improved PFS (HR: 0.33; *p* < 0.001) and persisted even with steroid use (HR: 0.38; *p* = 0.03). Indeed, steroids’ role and their effect on ICI therapy remain an evolving space with many discrepancies [72].

While there are no extensive RCTs data that discredit the association of irAEs with good clinical outcomes, a landmark analysis of a retrospective review of only 97 patients showed no difference in OS at 3 months in patients on single-agent ICI, even though the incidence of irAEs was associated with an OS (24.3 versus 5.3 months; HR: 2.75; 95% CI: 1.54–4.92; *p* < 0.001) for those with and without irAEs, respectively [32].

A systemic review of 16 studies reported that specific irAEs like mucositis, pneumonitis, myalgias and thyroid disorders are not statistically correlated with OS [73]. Moreover, potential bias and confounding factors were identified in the assumption that patients who develop irAEs remain on treatment longer and hence have a better outcome than their counterparts, and as such, could only be attributed to guarantee-time bias [74, 75].

Amongst the coterie of the larger studies looking at this association, the consensus is that there is potentially an association between the development of irAEs and OS, ORR and PFS in patients treated with ICIs, irrespective of disease site, type of ICI and irAEs spectrum. Conversely, higher degrees of toxicity grade (G3 or higher) led to a better ORR only but a worse OS [52]. More similar studies that investigated the association of irAEs and patients’ outcomes are shown in Table 2.

There is a need for a global initiative to develop an international registry on the reporting of irAEs with well-curated data to gauge the harm and the benefits of ICI agents and further understand if there is a clinically meaningful association between toxicities and survival benefits [76]. Also, an enhanced selection criterion to identify patients with advanced-stage cancers who will or will not benefit from ICI agents is essential, and the need to involve early primary palliative care for the potential non-responders. Patient education and a dedicated multi-disciplinary team are crucial for diagnosing and timely treatment of irAEs [77].

## Conclusions and perspectives

Despite all the controversy in this space, the general consensus is that irAEs are not a pre-requisite for ICI benefit but rather a positive heralding sign of clinical benefit. According to all the studies we reviewed, the association between irAEs and clinical outcomes remains disperse. While the objective response rate and DCR are mostly higher in those who develop irAEs, the OS and PFS data are dependent on the tumour type, degree of toxicities, affected spectrum of system/organs and inherent patient characteristics.

The development of irAEs appears to be more associated with both PD-1 and PD-L1 response and benefits as compared to anti-CTLA-4. There is a paucity of large well-powered studies to refute or confirm the exact benefit of the occurrence of irAEs during ICI therapy. Several questions remain unanswered, including the association between irAEs onset and the type of organ system toxicities, toxicity grading, ITBs, confounding comorbidities like autoimmune diseases, immune heterogeneity and patient intrinsic factors. Therefore, a harmonised global registry on the incidence of irAEs and their associated clinical outcomes is required to open a new era of immuno-oncology.

## Conflicts of interest

The authors declare that they have no conflicts of interest.

## Authors’ contributions

All authors substantially contributed to the conception, drafting and revising of the work and approved the final manuscript. All authors agree to be accountable for all aspects of the work in ensuring the accuracy and integrity of the work.

## Funding

No funding was received for this work.

## Disclosures

None.

## Figures and Tables

**Table 1.** List of approved ICIs.

International nonproprietary name	Type	Target	FDA or CNMPA indications	Year of approval
Ipilimumab	IgG1 mAb	CTLA-4	RCC, melanoma, lung cancer	FDA-2011
Pembrolizumab	IgGa4 mAb	PD-1	Melanoma, NSCLC and SCLC, SCCHN, cHL, primary large B-cell lymphoma, UC, GC and breast cancer	FDA-2014
Nivolumab	IgG4 mAb	PD-1	Metastatic NSCLC, UC, metastatic melanoma, advanced RCC, SCLC, SCCHN, HCC, metastatic CRC and cHL	FDA-2014
Ipilimumab & Nivolumab combination	IgG1 + IgG4mAb	CTLA-4 & PD-1	Melanoma, RCC, NSCLC and CRC	FDA-2015
Atezolizumab	IgG1 mAb	PD-L1	NSCLC, UC, breast cancer, melanoma, RCC, CRC and SCCHN	FDA-2016
Avelumab	IgG1 mAb	PD-L1	Merkel-cell carcinoma, RCC and NSCLC	FDA-2017
Durvalumab	IgG1 mAb	PD-L1	UC, NSCLC and SCLC	FDA-2017
Cemiplimab	IgG1 mAb	PD-1	Metastatic cutaneous squamous cell carcinoma and myeloma	FDA-2018
Toripalimab	IgG4 mAb	PD-1	Locally advanced or metastatic melanoma	CNMPA-2018
Sintilimab	IgG4 mAb	PD-1	Refractory cHL, HCC and NSCLC	CNMPA-2018
Camrelizumab	IgG4/k mAb	PD-1	cHL, metastatic HCC, metastatic esophageal cancer and advanced NSCLC	CNMPA-2019
Tislelizumab	IgG4 mAb	PD-1	Refractory cHL and UC	CNMPA-2019

CRC, Colorectal cancer; cHL, Classic Hodgkin lymphoma; HCC, Hepatocellular carcinoma; RCC, Renal cell carcinoma; SCCHN, Squamous cell carcinoma of the head and neck; NSCLC, Non-small cell lung cancer; SCLC, Small cell lung cancer; UC, Urothelial carcinoma; FDA: Food and Drug Administration; CNMPA, Chinese national medical product administration

**Table 2.** Studies comparing the association between irAEs and clinical outcomes.

Study	Cancer type	Sample size	ICI agent	Survival outcomes in patients with and without irAEs (OS, PFS)	RR in patients with versus without irAEs
Weber *et al* [41]	Melanoma	576	Nivolumab	PFS (no difference; HR not available)	48.6% versus 17.8%; *p* = < 0.001
Indini *et al* [51]	Melanoma	173	Nivolumab or pembrolizumab	OS (HR: 0.39; 95% CI: 0.18–0.81; *p* = 0.007) PFS (HR 0.47; 95% CI 0.26–0.86; p = .016)	ORR (HR: 1.95; 95% CI: 0.91–4.15; *p* < 0.082) DCR (HR: 1.98; 95% CI: 1.07–3.67; *p* < 0.029)
Baldini *et al* [78]	NSCLC	1959	Nivolumab	OS: 16.7 months (95% CI: 13.5–19.9) versus 9.4 (95% CI: 8.4–10.4); *p* < 0.00001 PFS: 6.0 months (95% CI: 4.9–7.1) versus 3.0 (95% CI: 2.8–3.2); *p* < 0.0001	RR: 27.2% versus 15.2%; *p* < 0.0001 DCR: 60.5% versus 40.2%; *p* < 0.0001
Shankar *et al* [79]	NSCLC	623	ICI monotherapy or in combination	OS (HR: 0.86; 95% CI: 0.66–1.12; *p* = 0 .26) and PFS (HR: 0.68; 95% CI: 0.55–0.85; *p* = 0.001)	Not available
Grangeon *et al* [80]	NSCLC	270	Anti-PD-L1 or anti-PD-1	OS (HR: 0.29; 95% CI: 0.18–0.46; *p* = 0.001) and PFS (HR: 0.42; 95% CI: 0.32–0.57; *p* = < 0.001)	22.9% versus 5.7%; *p* = < 0.0001) and DCR (76% versus 58%; *p* = < 0.001)
Ricciuti *et al* [81]	NSCLC	195	Nivolumab	OS (HR: 0.33; 95% CI: 0.23–0.47; *p* < 0.001) and PFS (HR: 0.41; 95% CI: 0.3–0.57; *p* < 0.001)	ORR (43.5% versus 10%; *p* < 0.001) and DCR (70.5% versus 18.1%; *p* < o.0001)
Vitale *et al* [82]	mRCC	167	Nivolumab	OS (20.1 months; HR: 0.38; 95% CI: 0.23–0.63) PFS (7.86 months; HR: 0.44; 95% CI: 0.29–0.66)	ORR (27.3% versus 13.7%; OR: 2.36; 95% CI: 1.03–5.44) DCR (68.8% versus 48%; OR: 2.4; 95% CI: 1.23–4.67)
Verzoni *et al* [83]	RCC	389	Nivolumab	OS (HR: 0.57; 95% CI: 0.35–0.93; *p* = 0.02)	Not available
Maher *et al* [84]	UC	1,747	Pembrolizumab or Atezolizumab	OS (HR: 0.53; 95% CI: 0.43–0.66)	Not available
Morales-Berera *et al* [85]	UC	52	ICI agents	OS (21.91 versus 6.47 months; *p* = 0.004)	DCR (79% versus 36.3%; *p* = 0.002)
Foster *et al* [67]	HNC	114	Anti-PD therapy	OS (12.5 versus 6.8 months; *p* = 0.0007) and PFS (6.9 versus 2.1 months; *p* = 0.0004)	ORR (30.6% versus 12.3%; *p* = 0.02)
Ando *et al* [86]	Advanced GC	108	Nivolumab or Pembrolizumab	OS (12.2 months; 95% CI = 3.8–NA) versus (3.5 months (95% CI: 2.9–5.1) PFS (3.9 months; 95% CI = 2.8–9.3) versus 1.8 months (95% CI = 1.4–-2.1)	28.5% versus 3%
Masuda *et al* [87]	GC	65	Nivolumab	OS (HR: 0.17; *p* < 0.001) and PFS (HR: 0.11; *p* < 0.001)	Not available
Das *et al* [61]	Gastrointestinal tumours	76	ICI monotherapy or in combination	OS (32.4 versus 8.5 months; *p* = 0.0036), PFS (32.4 versus 4.8 months; *p* = 0.0001)	Not available
Riudavets *et al* [88]	Solid tumours	178	Pembrolizumab, nivolumab and atezolizumab	OS (37.3 versus 7.8 months; *p* < 0.0001) and PFS (7.9 versus 2.6 months; *p* < 0.0001)	Not available
Matsuoka *et al* [89]	Across various tumours	280	Any ICI agent	OS (*p* < 0.01) and PFS (*p* < 0.01)	ORR (30.4% versus 12.7%; *p* < 0.01)

irAEs, Immune-related adverse events; ICI, Immune checkpoint inhibitors; OS, Overall survival; PFS, Progression-free survival; HR, Hazard ratio; CI, Confidence interval; ORR, Overall response rate; DCR, Disease control rate; NA, Not applicable; NSCLC, Non-small cell lung cancer; mRCC, Metastatic renal cell carcinoma; UC, Urothelial carcinoma; HNC, Head and neck cancer
